# Prolonged facemask wearing among hospital workers and dry eye – a mixed-methods study

**DOI:** 10.1186/s12886-023-03153-3

**Published:** 2023-10-19

**Authors:** Tianjing Li, Paul M. McCann, Sarah Wilting, Steve McNamara, Darren G. Gregory, Scott G. Hauswirth, Cristos Ifantides, Lorie Benning, Tamara A. Sequeira, Riaz Qureshi, Su-Hsun Liu, Melissa A. Clark, Ian J. Saldanha, Alison G. Abraham

**Affiliations:** 1 Department of Ophthalmology, University of Colorado Anschutz Medical Campus, Aurora, CO USA; 2 Department of Epidemiology, Colorado School of Public Health, Aurora, CO USA; 3 Department of Health Services, Policy, and Practice, Brown University School of Public Health, Providence, RI USA; 4 Department of Epidemiology, Johns Hopkins Bloomberg School of Public Health, Baltimore, MD USA; 5 Survey Research Center, Brown University School of Public Health, Providence, RI USA

**Keywords:** Dry eye, Hospital workers, Mixed-methods study

## Abstract

**Background:**

Prolonged facemask wearing may have negatively affected essential workers with dry eye. We conducted a mixed-methods study to examine and understand the associations of the ocular surface, periocular environment, and dry eye-related symptoms among hospital workers across the job spectrum with prolonged facemask use.

**Methods:**

We recruited clinical and non-clinical hospital workers with self-reported symptoms of dry eye and prolonged facemask use. We measured symptoms using the 5-item Dry Eye Questionnaire and the Ocular Surface Disease Index (OSDI). Objective ocular signs included corneal and conjunctival staining, fluorescein tear break up time (TBUT), meibography, tear film interferometry, and periocular humidity. We compared symptoms and signs across levels of periocular humidity, dry eye severity, facemask type, and job type. Participants with moderate or severe dry eye symptoms (OSDI > = 23) were invited for a semi-structured, one-on-one interview.

**Results:**

We enrolled 20 clinical and 21 non-clinical hospital workers: 27% were 40 years or older, 76% were female, 29% reported a race other than White, and 20% were Hispanic. Seventeen individuals participated in the semi-structured interviews. From the quantitative analyses, we found that 90% of participants reported worsened severity of dry eye at work due to facemasks. Although wearing facemasks resulted in higher periocular humidity levels compared with not wearing facemasks, 66% participants reported increased airflow over their eyes. Findings from the qualitative interviews supported the finding that use of facemasks worsened dry eye symptoms, especially when facemasks were not fitted around the nose. The data did not suggest that non-clinical hospital workers experienced a greater impact of dry eye than clinical workers.

**Conclusions:**

Healthcare providers and patients with dry eye should be educated about the discomfort and the ocular surface health risks associated with inadequately fitted facemasks. Wearing a fitted facemask with a pliable nose wire appears to mitigate the upward airflow.

**Supplementary Information:**

The online version contains supplementary material available at 10.1186/s12886-023-03153-3.

## Background

Dry eye is a multifactorial disease of the tear film and ocular surface that results in symptoms of discomfort and visual disturbance due to tear film instability and potential damage to the ocular surface, [1] all of which substantially impact patient quality of life. Dry eye is one of the most common reasons for patients to seek eye care, with an estimated prevalence of 8.1% in the U.S. population [2]. The societal cost of managing dry eye is also substantial, estimated to be $55.4 billion per year in 2011 [3]. Notably, dry eye medications are associated with the highest medication expenditures and out-of-pocket costs among all ocular medication classes [4].

During the COVID-19 pandemic, multiple studies found that prolonged facemask wearing may have negatively affected individuals with dry eye, particularly among essential workers [5–14]. The prevailing hypothesis is that the increased airflow directed upward from the facemask towards the eyes may accelerate tear film evaporation, leading to dryness and inflammation of the ocular surface. However, the findings of these studies were inconsistent (e.g., not all studies showed worsened Ocular Surface Disease Index), and none of them measured humidity levels in front of the eyes or sought opinions from patients regarding their experiences with facemasks.

Our recent work suggests that individuals from lower socioeconomic positions may be disproportionately affected by the burden of dry eye due to structural and economic inequalities [15]. These inequalities have likely been deepened in the context of the COVID-19 pandemic. Further, we have shown that work efficiency during the COVID-19 pandemic was significantly influenced by the severity of dry eye symptoms, workplace flexibility, and access to treatments or interventions that could alleviate symptoms [16].

Here we report on a mixed-methods study conducted to examine and understand the associations of the ocular surface, periocular environment, and dry eye-related symptoms among a sample of hospital workers across the job spectrum exposed to regular and prolonged facemask use. A mixed-methods study strategically collects and integrates both quantitative and qualitative data drawing on the strengths of each and providing more nuanced interpretations of the data and a broader applicability of small samples [17]. We hypothesized that the impact of facemask wearing on dry eye symptoms and signs among essential workers may vary by periocular humidity level, facemask type, whether the facemask is fitted and worn properly, and across different socioeconomic groups, including those with limited access to resources and support.

## Methods

### Study setting and eligibility criteria

This single-site study was conducted at the Sue Anschutz-Rodgers Eye Center, University of Colorado Anschutz Medical Campus, Aurora, Colorado (IRB protocol number: 21-2541). We recruited non-clinical and clinical hospital workers who were 18 years or older with self-reported symptoms of dry eye and who wore facemasks for at least 80% of the time during shifts lasting 6 h or longer. We excluded individuals with other active ocular surface diseases, such as conjunctivitis, abrasion of the cornea or conjunctiva, recurrent corneal erosion syndrome, episcleritis, inflamed pterygium, tumor in the eye, and infectious keratitis. We also excluded persons who had ocular surgeries performed on either eye during the COVID-19 pandemic (since March 1, 2020). Eligibility was assessed initially by the prospective participants who completed a secure, online screening form developed and deployed using REDCap (Research Electronic Data Capture). The study coordinator, a trained optometrist, reached out to the potentially eligible individuals and inquired about history of ocular surface diseases and ocular surgeries before enrolling the prospective participant and scheduling the measurement visit.

### Measurements

The measurement visit was scheduled at the end of a shift. Participants were instructed to wear the same type of facemask that they had worn during a typical hospital shift. During the measurement visit, the study coordinator obtained written informed consent from participants. We collected various characteristics about each participant, including age, sex at birth, job type, type of facemask used at work, average duration of facemask use at work, history and duration of dry eye disease, risk factors for dry eye (e.g., history of smoking, wearing of contact lenses or glasses, computer use time), and treatments used for dry eye.

To measure dry eye symptoms, we used two validated, self-administered instruments: the 5-Item Dry Eye Questionnaire (DEQ-5) and the Ocular Surface Disease Index (OSDI). To measure clinical features of the ocular surface, the study coordinator performed five assessments in an order of least to most invasive: (i) humidity in front of each eye with and without facemask using a custom-built hygrometer (Hu-Med Technologies, Inc. Denver, Colorado), (ii) lipid layer interferometry (TearScience® LipiView® II Ocular Surface Interferometer with Dynamic Meibomian Imaging (Johnson & Johnson Vision, California USA)), (iii) fluorescein tear film break up time (TBUT), (iv) corneal and conjunctival staining, which were assessed by applying fluorescein sodium and lissamine green ophthalmic strips to the inferior conjunctiva, and (v) meibography (TearScience® LipiView® II Ocular Surface Interferometer with Dynamic Meibomian Imaging, Johnson & Johnson Vision, California USA). All data were collected electronically using REDCap on tablet devices. We compensated participants $50 for their time spent on the visit.

### Qualitative interviews

Participants with moderate or severe dry eye, defined as OSDI of 23 or greater, were eligible for a follow-up, semi-structured, one-on-one telephone interview. The initial interview guide was developed by the study team and iteratively modified based on experiences and participant feedback during pilot interviews. During the pilot interviews, participants with mild dry symptoms did not report worsening symptoms from facemask use and described feeling that they had very little to contribute to the interview. Hence, we limited interviews to participants with moderate/severe dry eye. Trained qualitative researchers at the Brown University School of Public Health Survey Research Center interviewed those who agreed to participate. We asked participants to describe their experiences with facemasks, as well as how and why facemasks may have exacerbated their dry eye symptoms. The interview questions are listed in the Appendix. With participant consent, interviews were recorded and professionally transcribed for data analysis. We compensated participants $50 for their time spent in the interview.

### Analysis

For the quantitative component of the study, the target sample size was 40 participants with equal representation (n = 10) within each of the four categories: (1) clinical workers (e.g., doctors, nurses, therapists, pharmacists, technicians) with moderate/severe dry eye symptoms (i.e., OSDI score of 23 or greater); (2) non-clinical workers (e.g., clerical staff, janitorial staff, food services staff, environmental services staff) with moderate/severe dry eye symptoms; (3) clinical workers with normal/mild dry eye symptoms (i.e., OSDI score between 0 and 22); and (4) non-clinical workers with normal/mild dry eye symptoms. We did not conduct any formal power calculations to reach this target sample size because the goal of the study was descriptive and hypothesis generating. For the qualitative component of the study, only participants with moderate/severe dry eye symptoms were invited to participate.

We summarized the distribution of each measurement using, as appropriate, medians and interquartile ranges (IQRs) for continuous measures and proportions for categorical measures. We generated a heatmap to visualize the patterns of measurements. We compared groups using small sample statistics: Fisher’s exact tests for categorical outcomes and Kruskal-Wallis tests for continuous outcomes. All associations reported are bivariable, that is, not adjusted for the influence from other factors due to the limitations of the small sample size. We considered p-value less than 0.05 as statistically significant; however, we caution interpretation of p-value as the study is exploratory in nature. We analyzed the quantitative data using SAS version 9.4.

For the qualitative component, we examined data from interviewer recordings, transcripts, and notes taken by the interviewers using a template style approach [18]. The initial version of the coding template was based on themes identified in the pilot interviews. Data were sorted into topics by two coders and then analysed by three members of the study team to identify themes within and across interviews.

## Results

### Sample characteristics

Between March 2022 and July 2022, a total of 41 participants, including 20 clinical (49%) and 21 non-clinical hospital workers (51%) were enrolled in the study. Of the 22 participants who were classified as having moderate/severe dry eye symptoms using OSDI, 17 (77%) participated in the semi-structured, one-on-one interviews conducted between July 2022 and August 2022. The semi-structured interviews were 17 min on average in length (range: 7–34 min). Table 1 displays the characteristics of the study participants in the full sample (n = 41) and in the qualitative interview sample (n = 17).

**Table 1:** Characteristics of study participants

	Full Sample (n = 41)		Participated in qualitative interviews (moderate to severe symptoms; n = 17)		
Characteristics	n	%	n	%	
**Job type**					
Clinical	20	48.8	9	52.9	
Non-clinical	21	51.2	8	47.1	
**Age group**					
18–29	16	39.0	7	41.2	
30–39	14	34.2	4	23.5	
40–49	8	19.5	5	29.4	
60–69	3	7.3	1	5.9	
**Sex**					
Female	31	75.6	15	88.2	
Male	10	24.4	2	11.8	
**Race**					
White	26	63.4	11	64.7	
Black or African American	3	7.3	1	5.9	
American Indian or Alaska Native	2	4.9	1	5.9	
Asian or Pacific Islander	6	14.6	3	17.7	
Multi-race	1	2.4	0	0.0	
Refused	3	7.3	1	5.9	
**Ethnicity**					
Non-Hispanic	33	80.5	13	76.5	
Hispanic	8	19.5	4	23.5	
Household income before tax*					
<$50,000	13	33.3	4	25.0	
$50,000-$99,999	16	41.0	9	56.3	
≥$100,000	10	25.6	3	18.8	
**Current smoker**					
No	38	92.7	16	94.1	
Yes	3	7.3	1	5.9	
**Contact lenses wear at least 4 days/week**					
No	39	95.1	16	94.1	
Yes	2	4.9	1	5.9	
**Spectacles wear at least 4 days/week**					
No	15	36.6	7	41.2	
Yes	26	63.4	10	58.8	
**Average screen time**					
0–4 h	8	19.5	3	17.7	
5–7 h	15	36.6	5	29.4	
8 + hours	18	43.9	9	52.9	
**Screen time most recent shift**					
0–4 h	8	19.5	3	17.7	
5–7 h	18	43.9	7	41.2	
8 + hours	15	36.6	7	41.2	
**Dry eye diagnosis**					
No	20	48.8	8	47.1	
Yes	17	41.5	7	41.2	
Unsure	4	9.8	2	11.8	
**Dry eye treatment**					
Over the counter (OTC) only	12	29.3	3	17.7	
OTC plus other	16	39.0	9	52.9	
None	13	31.7	5	29.4	
**Treatment success**					
Successful	16	39.0	5	29.4	
Not successful	12	29.3	7	41.2	
No treatment	13	31.7	5	29.4	
**Facemask type**					
N95	5	12.2	4	23.5	
Surgical or cloth	36	87.8	13	76.5	
**Facemask worn properly**					
No	7	17.1	3	17.7	
Yes	34	82.9	14	82.4	
Mask wearing duration at the most recently concluded shift**					
< 8 h	16	40.0	7	41.2	
8 h	12	30.0	5	29.4	
> 8 h	12	30.0	5	29.4	
**Changes during COVID**					
**Facemask wearing duration**					
Decreased	6	14.6	2	11.8	
No change	17	41.5	7	41.2	
Increased	18	43.9	8	47.1	
**Airflow blown over eyes**					
Decreased	4	9.8	1	5.9	
No change	7	17.1	4	23.5	
Increased	27	65.9	12	70.6	
Not sure	3	7.3	0	0.0	
**Perceived change in severity of dry eye at work**					
Decreased	0	0.0	0	0.0	
No change	4	9.8	0	0.0	
Increased	36	87.8	17	100.0	
Not sure	1	2.4	0	0.0	
**Perceived change in severity of dry eye NOT at work**					
Decreased	5	12.2	2	11.8	
No change	18	43.9	6	35.3	
Increased	18	43.9	9	52.9	

*Data missing from two participants

** Data missing from one participant

Of the total participants, 27% (n = 11) were aged 40 years or older, 76% (n = 31) were female, 29% (n = 12) reported a race other than White, and 20% (n = 8) were Hispanic (Table 1). A third (n = 13) of participants reported household income less than $50,000, 41% (n = 16) reported household income between U.S. $50,000 to $99,999, and the remaining 26% (n = 10) reported household income of $100,000 or more. Modifiable dry eye risk factors were low, with only 7% (n = 3) participants being current smokers and 5% (n = 2) wearing contact lenses for at least 4 days a week. 63% (n = 26) of participants reported wearing spectacles for at least 4 days a week. Most participants (81%, n = 33) reported using screens for 5 h or longer on average as well as during the most recent shift. The study included both participants with and without a clinician-confirmed dry eye diagnosis, who were being treated for dry eye, and who found the treatment to be successful. During the study visit, 88% (n = 36) of participants wore either surgical or cloth masks, with 83% (n = 34) wearing them properly, defined as nasal bridge sealed from the frontal, lateral, and transverse views. 60% (n = 24) of participants reported wearing facemasks 8 h or more during the most recently concluded shift. Nearly half of the participants (44%, n = 18) reported an increase in facemask-wearing duration during the COVID-19 pandemic, while two-thirds (66%, n = 27) reported an increase in airflow over the eyes when wearing a facemask. Nearly 90% (n = 36) of participants reported an increase during the COVID-19 pandemic in the perceived severity of dry eye symptoms while at work and 44% (n = 18) reported an increase while not at work.

Using the OSDI scores, approximately half of the participants (46%, n = 19) were classified as having no/mild dry eye symptoms; the remaining 54% (n = 22) were categorized as having moderate/severe dry eye symptoms (Table 2). This was consistent with the classification based on the DEQ-5 scores, which showed a similar number of participants with moderate/severe dry eye symptoms, with 22 (54%) scoring 12 or more points on the DEQ-5. The overlap between OSDI and DEQ-5 for moderate/severe symptoms was 18 (81%) participants.

**Table 2:** Associations between participant characteristics and dry eye symptoms and signs

	5-Item Dry Eye Questionnaire			Ocular Surface Disease Index			Corneal Staining			Conjunctival Staining			TBUT (worse eye)		LLT (worse eye)	
	Not severe	Severe	P-value	Normal/Mild	Moderate/Severe	P-value	No	Yes	P-value	No	Yes	P-value	Median (IQR)	P-value	Median (IQR)	P-value
**Job type**			0.35			0.76			0.16			1.00		0.43		0.68
Clinical	11 (55%)	9 (45%)		10 (50%)	10 (50%)		7 (35%)	13 (65%)		6 (30%)	14 (70%)		3.65 (2.29–5.19)		69 (59–83)	
Non-clinical	8 (38%)	13 (62%)		9 (43%)	12 (57%)		3 (14%)	18 (86%)		7 (33%)	14 (67%)		2.56 (1.85–4.64)		68 (51–80)	
**Age group**			0.07			0.20			0.14			1.00		0.01		0.55
< 30	8 (50%)	8 (50%)		5 (31%)	11 (69%)		3 (19%)	13 (81%)		5 (31%)	11 (69%)		2.90 (2.20–6.93)		68 (54–86)	
30–39	9 (64%)	5 (36%)		9 (64%)	5 (36%)		6 (43%)	8 (57%)		4 (29%)	10 (71%)		4.82 (3.32–5.48)		64 (56–72)	
≥ 40	2 (18%)	9 (82%)		5 (45%)	6 (55%)		1 (9%)	10 (91%)		4 (36%)	7 (64%)		2.33 (1.40–2.67)		74 (48–94)	
**Sex**			0.03			0.03			0.40			0.70		0.30		0.01
Female	11 (35%)	20 (65%)		11 (35%)	20 (65%)		9 (29%)	22 (71%)		9 (29%)	22 (71%)		2.56 (2.10–4.89)		72 (57–90)	
Male	8 (80%)	2 (20%)		8 (80%)	2 (20%)		1 (10%)	9 (90%)		4 (40%)	6 (60%)		4.69 (1.83–6.17)		57 (48–64)	
**Race**			0.73			0.73			0.22			0.27		0.15		0.13
White	13 (50%)	13 (50%)		13 (50%)	13 (50%)		8 (31%)	18 (69%)		10 (38%)	16 (62%)		3.46 (2.34–5.48)		71 (64–82)	
Non-white	5 (42%)	7 (58%)		5 (42%)	7 (58%)		1 (8%)	11 (92%)		2 (17%)	10 (83%)		2.38 (1.84–4.17)		60 (50–75)	
**Ethnicity**			1.00			0.70			0.65			1.00		0.58		0.74
Non-Hispanic	15 (45%)	18 (55%)		16 (48%)	17 (52%)		9 (27%)	24 (73%)		11 (33%)	22 (67%)		3.32 (2.29–4.98)		68 (57–81)	
Hispanic	4 (50%)	4 (50%)		3 (38%)	5 (63%)		1 (13%)	7 (88%)		2 (25%)	6 (75%)		2.48 (1.81–5.06)		61 (49–90)	
**Household income**			0.71			0.34			0.44			0.53		0.06		0.41
<$50,000	7 (54%)	6 (46%)		5 (38%)	8 (62%)		4 (31%)	9 (69%)		6 (46%)	7 (54%)		4.64 (2.34–7.09)		64 (51–80)	
$50,000-$99,999	6 (38%)	10 (63%)		7 (44%)	9 (56%)		2 (13%)	14 (88%)		4 (25%)	12 (75%)		2.31 (1.84–3.19)		71 (64–87)	
≥$100,000	5 (50%)	5 (50%)		7 (70%)	3 (30%)		3 (30%)	7 (70%)		3 (30%)	7 (70%)		3.82 (2.56–5.40)		65 (46–76)	
**Current smoker**			0.24			1.00			0.14			0.23		0.34		0.92
No	19 (50%)	19 (50%)		18 (47%)	20 (53%)		8 (21%)	30 (79%)		11 (29%)	27 (71%)		3.19 (2.29–4.98)		68 (54–81)	
Yes	0 (0%)	3 (100%)		1 (33%)	2 (67%)		2 (67%)	1 (33%)		2 (67%)	1 (33%)		1.51 (1.35–9.16)		74 (37–82)	
**Average screen time**			0.003			0.12			0.90			0.52		0.17		0.03
0–4 h	6 (75%)	2 (25%)		5 (63%)	3 (38%)		2 (25%)	6 (75%)		3 (38%)	5 (63%)		4.27 (3.65–6.04)		60 (51–92)	
5–7 h	10 (67%)	5 (33%)		9 (60%)	6 (40%)		3 (20%)	12 (80%)		3 (20%)	12 (80%)		3.32 (2.29–4.98)		64 (49–70)	
8 + hours	3 (17%)	15 (83%)		5 (28%)	13 (72%)		5 (28%)	13 (72%)		7 (39%)	11 (61%)		2.44 (1.85–5.40)		75 (68–91)	
**Screen time most recent shift**			0.0003			0.18			1.00			0.61		0.15		0.02
0–4 h	5 (63%)	3 (38%)		5 (63%)	3 (38%)		2 (25%)	6 (75%)		3 (38%)	5 (63%)		4.27 (2.61–6.04)		59 (43–92)	
5–7 h	13 (72%)	5 (28%)		10 (56%)	8 (44%)		4 (22%)	14 (78%)		4 (22%)	14 (78%)		3.34 (2.44–4.98)		64 (51–70)	
8 + hours	1 (7%)	14 (93%)		4 (27%)	11 (73%)		4 (27%)	11 (73%)		6 (40%)	9 (60%)		2.33 (1.40–5.40)		76 (70–94)	
**Dry eye diagnosis**			0.75			0.75			1.00			0.04		0.64		0.40
No	12 (50%)	12 (50%)		12 (50%)	12 (50%)		6 (25%)	18 (75%)		11 (46%)	13 (54%)		2.94 (2.08–6.12)		67 (53–77)	
Yes	7 (41%)	10 (59%)		7 (41%)	10 (59%)		4 (24%)	13 (76%)		2 (12%)	15 (88%)		3.05 (2.10–4.64)		72 (56–82)	
**Dry eye treatment**			1.00			0.28			0.26			0.91		0.91		0.30
OTC only	6 (50%)	6 (50%)		8 (67%)	4 (33%)		4 (33%)	8 (67%)		3 (25%)	9 (75%)		3.55 (2.01–5.58)		62 (50–70)	
OTC plus other	7 (44%)	9 (56%)		6 (38%)	10 (63%)		5 (31%)	11 (69%)		6 (38%)	10 (63%)		2.86 (2.08–4.62)		73 (53–87)	
None	6 (46%)	7 (54%)		5 (38%)	8 (62%)		1 (8%)	12 (92%)		4 (31%)	9 (69%)		2.44 (2.10–4.74)		71 (63–80)	
**Treatment success**			0.16			0.68			0.08			1.00		0.40		0.48
Successful	10 (63%)	6 (38%)		9 (56%)	7 (44%)		7 (44%)	9 (56%)		5 (31%)	11 (69%)		3.82 (2.43–5.79)		64 (53–73)	
Not successful	3 (25%)	9 (75%)		5 (42%)	7 (58%)		2 (17%)	10 (83%)		4 (33%)	8 (67%)		2.55 (1.85–3.38)		73 (48–96)	
No treatment	6 (46%)	7 (54%)		5 (38%)	8 (62%)		1 (8%)	12 (92%)		4 (31%)	9 (69%)		2.44 (2.10–4.74)		71 (63–80)	
**Facemask type**			0.65			0.05			1.00			1.00		0.65		0.21
N95	3 (60%)	2 (40%)		0 (0%)	5 (100%)		1 (20%)	4 (80%)		1 (20%)	4 (80%)		2.56 (2.34–3.37)		56 (49–68)	
Surgical or cloth	16 (44%)	20 (56%)		19 (53%)	17 (47%)		9 (25%)	27 (75%)		12 (33%)	24 (67%)		3.19 (1.86–5.44)		70 (56–83)	
**Facemask worn properly**			1.00			0.68			1.00			0.40		0.63		0.46
No	3 (43%)	4 (57%)		4 (57%)	3 (43%)		2 (29%)	5 (71%)		1 (14%)	6 (86%)		2.67 (1.51–3.85)		54 (46–94)	
Yes	16 (47%)	18 (53%)		15 (44%)	19 (56%)		8 (24%)	26 (76%)		12 (35%)	22 (65%)		3.19 (2.10–5.40)		69 (57–81)	
Facemask wearing duration at the most recently concluded shift*																
			0.37			0.45			0.60			0.21		0.74		0.24
< 8 h	10 (63%)	6 (38%)		8 (50%)	8 (50%)		4 (25%)	12 (75%)		4 (25%)	12 (75%)		3.34 (2.50–4.24)		64 (49–79)	
8 h	5 (42%)	7 (58%)		7 (58%)	5 (42%)		2 (17%)	10 (83%)		2 (17%)	10 (83%)		2.31 (1.98–6.08)		66 (53–72)	
> 8 h	4 (33%)	8 (67%)		4 (33%)	8 (67%)		4 (33%)	8 (67%)		6 (50%)	6 (50%)		2.60 (1.54–5.79)		75 (63–96)	
**Periocular humidity**																
Masked			1.00			0.12			0.28			0.74		0.62		0.07
≤Median	10 (48%)	11 (52%)		7 (33%)	14 (67%)		7 (33%)	14 (67%)		6 (29%)	15 (71%)		2.56 (2.10–3.85)		64 (49–71)	
>Median	9 (45%)	11 (55%)		12 (60%)	8 (40%)		3 (15%)	17 (85%)		7 (35%)	13 (65%)		3.51 (2.07–5.83)		73 (62–91)	
Maskless			0.76			0.12			0.72			0.51		0.51		0.22
≤Median	9 (43%)	12 (57%)		7 (33%)	14 (67%)		6 (29%)	15 (71%)		8 (38%)	13 (62%)		3.37 (2.33–5.40)		65 (49–77)	
>Median	10 (50%)	10 (50%)		12 (60%)	8 (40%)		4 (20%)	16 (80%)		5 (25%)	15 (75%)		2.81 (1.84–4.94)		71 (62–87)	

P-values come from Fisher’s exact tests for categorical characteristics and from Kruskall-Wallis tests for comparison of medians

OTC: Over the counter

TBUT: tear breakup time; unit second

LLT: lipid layer thickness; unit nm

*Data missing from one participant

Corneal and conjunctival staining were present in 31 (76%) and 28 (68%) participants, respectively. The median TBUT, based on the worse eye of each participant, was 3.05 s (IQR 2.10 to 4.98), and the median Arita grade of Meibography was 2 (IQR 2 to 3). The median periocular humidity was 29.0% (IQR 21.0–35.0%) when wearing facemasks and 19.4% (IQR 14.3–26.8%) without facemasks.

In terms of bivariable associations (Table 2), female sex and longer screen time were associated with more severe dry eye symptoms, as defined by DEQ-5 scores. When OSDI scores were used to define the severity of dry eye symptoms, a similar association was observed for female sex but not for screen time. Patient-reported symptom scores were worse when treatment was unsuccessful or when duration of facemask wearing was longer. Among participants with unsuccessful treatment (based on participant self-report), 83% had corneal staining. Dry eye diagnosis was associated with greater conjunctival staining. Although the TBUT score (worse eye) was only statistically significantly associated with age, scores were generally lower (and worse) among participants identified as non-White, Hispanic, female, smokers, middle-income bracket, and non-clinical workers. The score was also lower with increased screen time during the most recent shift and longer facemask duration. The lipid layer thickness measures were statistically significantly lower among males and were negatively associated increased screen time. Although not statistically significant, lipid layer thickness measures were higher among those with longer facemask duration, surgical facemask wear, and increased periocular humidity.

Regarding our hypotheses, the data do not suggest that periocular humidity level and proper facemask fit were associated with variations in OSDI scores, DEQ-5 scores, corneal staining, conjunctival staining, TBUT scores, or lipid layer thickness. All five N95 wearers were classified as having moderate/severe dry eye symptoms, as compared with 47% (n = 17) of surgical or cloth mask wearers. Furthermore, the data do not suggest that non-clinical hospital workers experienced a greater impact of dry eye than clinical workers.

**Fig. 1:**
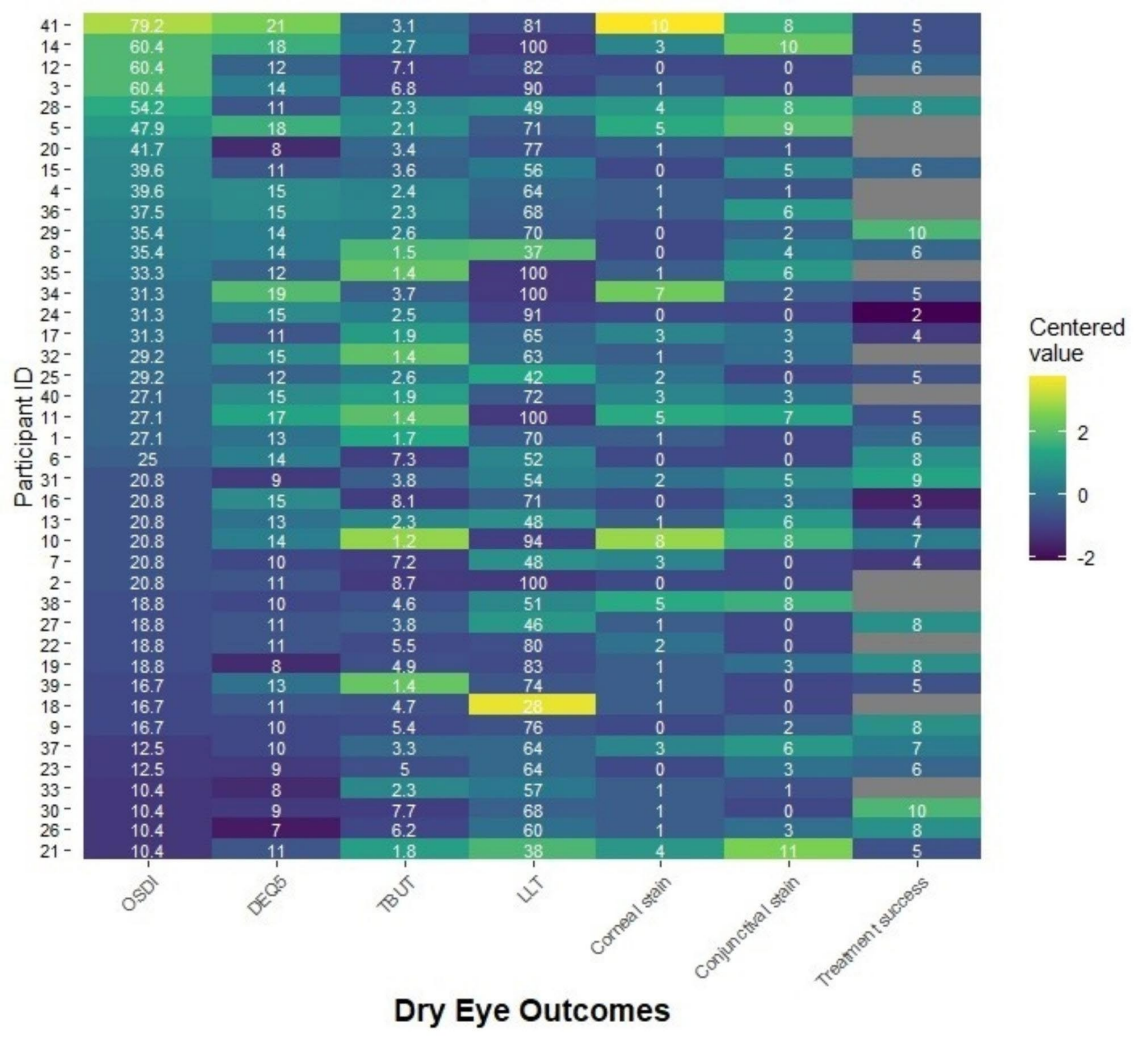
Measures of dry eye symptoms and signs, ordered by decreasing ocular surface disease index score. OSDI: ocular surface disease index. DEQ-5: 5-item dry eye questionnaire. TBUT: tear film breakup time; unit second. LLT: lipid layer thickness; unit nm

Figure 1 presents a visualization of the measurements on dry eye symptoms and ocular surface in a heatmap, sorted by decreasing OSDI score. This visualization did not produce any new insights to the relationships between variables. Figure 1 includes the actual scores from all clinical assessments as white text within each cell. The color of the cells represents a centred (i.e., Z-score standardized) value for that cell based on the assessment’s distribution, such that “bright yellow” and “dark blue” respectively represent “worse” and “better” outcomes for all measures. There appeared to be some correlation between the OSDI and DEQ-5 scores (correlation coefficient = 0.60) and possible relationships between increasing TBUT with decreasing symptoms and increasing lipid layer thickness with increasing symptoms. There did not appear to be any patterns with staining, nor perceived treatment success among those who had tried treatments.

Of the participants eligible for the one-on-one interviews, compared to those who were not interviewed (n = 5), those who were interviewed (n = 17) were more likely to hold clinical hospital positions (53% vs. 20%), be 40 years of age or older (59% vs. 0%), identify as white or Asian/Pacific Islander (82% vs. 40%), report incomes of $50,000 or more (75% vs. 0%), report average screen times of less than 8 h (47% vs. 20%), have tried dry eye treatments in addition to over the counter medications (53% vs. 20%), report dry eye treatment as unsuccessful (41% vs. 0%), and report a facemask less than 8 h during their most recently concluded shift (41% vs. 25%).

Of the 17 individuals who participated in the one-on-one interviews, all but one reported that their dry eye had worsened when wearing a facemask. Seven reported that their dry eye was “significantly”, “noticeably”, or “much” worse. The specific symptoms they noted included dryness, burning sensation, grittiness, irritation, itching, foreign body sensation, and blurred vision. When asked whether a particular type of facemask made their dry eye symptoms worse, the responses were mixed: six participants reported that the surgical mask was worse, three reported that the N95 was worse, four reported no difference among facemask types, and the remaining participants described “one that are not fitted”, “not having a bendable nose wire”, or “thicker masks” as worse for them.

Regarding explanations, most participants reported that surgical masks had more gaps and air leaks around the nose, and that N95 masks offered a better seal around the face as exemplified by the following quotes:

*“The N95 does a job of keeping your nose in, so there’s limited air that goes through. And with the surgical masks, there’s more pockets. Even when you tighten it on the nose, more air can come through when you’re breathing for some reason.”* (Participant 1, severe dry eye symptoms).

*“I think the surgical mask seems to be the worst one because I feel like there are more leaks. Versus the N95 tends to seal around your face more.”* (Participant 8, severe dry eye symptoms).

*“You know how there’s the metal piece. You can shape it to your face. And then on the inside, there’s a foam piece [in one type of N95] that really helps reduce the airflow. So, it’s not a 100%, but that’s pretty much the only mask I can wear to make it through an entire day of masking. A surgical face mask… really just doesn’t sit against your face all at all, nasally and top of your cheeks, it just drives air straight into my eyes.* (Participant 4, severe dry eye symptoms)

*“The surgical definitely made the dry eye symptoms worse. Seems like the better seal on the N95 helps a little bit, but it’s still a lot of heat trapped beneath my glasses and stuff throughout the day”.* (Participant 12, severe dry eye symptoms)

The three participants who reported that the N95 mask made their dry eye symptoms worse described their experiences the following ways:

*“I can say N95 [made symptoms worse] because with the surgical mask, there’s some gap in between the mask and my cheeks as well, so the air can escape from that. But in case of N95, the only area that the carbon dioxide that I breathe out can escape from the area between my nose and my masks, so I can say N95 is much more difficult for me.”* (Participant 13, moderate dry eye symptoms).

*“Every time I would breathe, it was really tight enough everywhere else, but it would shoot up on the side of my nose, every time I would breathe, it’d shoot up towards my eye*.” (Participant 5, moderate dry eye symptoms).

*“I think the N95 is the worst one for dry eyes. And when I wear surgical masks… I don’t wear them as often as I do the N95 mask, but I do think that they’re better for my eyes. It’s more of a lightweight mask, and I don’t feel them impounding on my dry eyes*.” (Participant 7, moderate dry eye symptoms).

In general, participants reported that N95 masks fit on the nose better than surgical masks. Dry eye symptoms seemed to have affected the decisions of four participants regarding which type of mask they generally wore but not the decisions of the other participants. Of the participants for whom dry eye symptoms did not affect the decision of what type of facemask to wear, four did not have a choice and were required to wear an N95 mask.

## Discussion

This mixed-methods study found that nearly all participants reported worsened severity of dry eye at work since the introduction of mandatory facemask wearing during the COVID-19 pandemic. Although wearing facemasks resulted in higher periocular humidity levels compared with not wearing facemasks, two-thirds of participants also reported increased airflow over their eyes. While TBUT score (worse eye) showed a statistically significantly association with age, participants identified as non-White, Hispanic, female, smokers, in the middle-income bracket, and non-clinical workers generally exhibited lower (and worse) scores. Findings from the qualitative interviews supported the finding that use of facemasks had worsened dry eye symptoms, especially when facemasks are not fitted around the nose to seal off the upward airflow in the direction of the eye. Finally, the data do not suggest that non-clinical hospital workers experienced a greater impact of dry eye than clinical workers.

There are several potential reasons why the data did not support our primary and secondary hypotheses. First, the study was cross-sectional, and the sample size was relatively small given the exploratory nature of the study. Additionally, the number of participants wearing N95 masks was even smaller, which limited our ability to explore the potential effects of this facemask type on our outcomes of interest. Although the periocular humidity was higher when facemasks were worn, it is possible that the upward airflow may have an independent effect on tear evaporation. We hypothesize that the suspected increased air flow across the cornea caused by facemask wear outweighed the benefit of the higher water vapor (and therefore higher periocular relative humidity) from the participant’ exhaled breath. Previous literature has shown that people who suffer from evaporative dry eye could show a greater tear evaporation rate at 40% relative humidity compared with people without dry eye. However, at a relative humidity of 70%, the tear evaporative rate declines to zero in both groups [19]. Although a periocular hygrometer was valuable to investigate periocular relative humidity in the context of facemask wear, future studies should include micro air flow meters to allow for quantitative comparisons of patient symptomatology. Finally, other factors, such as screen time and dry eye treatment were not accounted for in our analysis, which may have obscured potential associations. It is suggested that during the COVID pandemic, increased screen time and digital device use may have exacerbated signs and symptoms of dry eye [14].

Our study confirmed the poor correlation between self-reported dry eye symptoms and ocular surface measures noted in many other studies [20, 21]. We also found an incoherent relationship between lipid layer thickness measures and other dry eye parameters, which has been previously reported [22, 23]. Caution should be exercised when interpretating lipid layer thickness data for several reasons. First, although the device measures interferometric grading between 10 and 240 nm, the output parameter range is 80 nm (20 – 100 nm), with a measurement accuracy of +/-10 nm (LipiView II Operating Specifications). Second, the quality of the meibum, which we did not measure in this study, may have a more profound effect than the volume of lipid in the tear film. Third, because the primary force moving meibum from within the gland to the tear film is the blink, [24] increased blinking frequency, force, or other simple actions, such as a brief eye rub, may create greater variability in this measurement.

We interviewed 77% of eligible participants for the one-on-one interviews. Eligible individuals not interviewed were more likely to hold non-clinical hospital positions, be younger, to spend more hours per day on screens, and to report that they had never been treated for dry eye symptoms or the treatment had been successful. It is unclear whether additional novel information would have been obtained if additional interviews had been conducted with these individuals.

Our study had several strengths. First, we utilized a mixed-methods design that incorporated the lived experiences of participants. By adding qualitative data to our quantitative data, we were able to deepen and enrich our interpretation of why dry eye symptoms were worsened due to facemask wear despite increased periocular humidity. Furthermore, we purposely recruited a diverse group of hospital workers to capture the full spectrum of job factors that may exacerbate or mitigate facemask-related dry eye symptom burden and as a surrogate for socioeconomic position. It is important to note that the lack of demonstrated differences between these two groups should not be interpreted as ‘evidence of no difference.‘ To provide more definite answers, we encourage future studies with large enough sample sizes to evaluate specific hypotheses regarding potential relationships between different aspects of social determinants and dry eye.

## Conclusions

In conclusion, our study found ocular irritation and dryness among both non-clinical and clinical hospital workers who wore facemasks for extended periods. Despite increased periocular humidity when wearing facemasks, the upward airflow may worsen dry eye symptoms. Healthcare providers and patients with dry eye should be educated about the discomfort and the ocular surface health risks associated with inadequately fitted facemasks. From a behavioral modification perspective, wearing fitted facemasks with a pliable nose wire may mitigate the upward airflow and reduce dry eye symptoms.

### Electronic supplementary material

Below is the link to the electronic supplementary material.

Supplementary Material 1

## Data Availability

The datasets generated and analyzed during this study are available from the corresponding author on reasonable request.
