# Supplementary material for: Prolonged facemask wearing among hospital workers and dry eye – a mixed-methods study
Source: BMC Ophthalmol. 2023 Oct 19;23:420. doi: 10.1186/s12886-023-03153-3 (PMC10588155; doi:10.1186/s12886-023-03153-3)
Supplement: Supplementary file 1 — Supplementary Material 1 [file 12886_2023_3153_MOESM1_ESM.docx]

Appendix. Semi-structured interview questions

1. To what extent does your dry eye worsened when wearing a mask?
2. What type(s) of masks do you generally wear?
3. Are there any particular types of masks that make your dry eye symptoms worsen?
4. To what extent do dry eye symptoms affect your decisions about which types of masks you generally wear?
5. What specific dry eye symptoms worsen with *[fill in response from #3]*?
6. Why do you think that this kind of mask makes your dry eye symptoms worse?
7. How well would you say that this type of facemask fits on your nose?
8. *Repeat 4-6 for each type of mask endorsed in #3.*
